# Intra-arterial Chemotherapy in Patients With Metastatic Breast Cancer: A Scoping Review

**DOI:** 10.7759/cureus.58846

**Published:** 2024-04-23

**Authors:** Mahi Basra, Hemangi Patel, Alejandro Biglione

**Affiliations:** 1 Osteopathic Medicine, Nova Southeastern University Dr. Kiran C. Patel College of Osteopathic Medicine, Clearwater, USA; 2 Sports Medicine, Nova Southeastern University Dr. Kiran C. Patel College of Osteopathic Medicine, Fort Lauderdale, USA; 3 Internal Medicine, Wellington Regional Medical Center, Wellington, USA

**Keywords:** breast metastases, breast cancer, metastatic breast cancer, hepatic intra-arterial, intra-arterial chemotherapy

## Abstract

Breast cancer is a common malignancy in women, and the survival rate for this cancer is low once it metastasized. Currently, chemotherapy is the first-line treatment for metastatic breast cancer (MBC). However, when liver metastases (LM) are present, the response to chemotherapy is poor. Regional intra-arterial chemotherapy (RIAC) delivers a high concentration of anticancer drugs to the malignant tissue, which improves the survival rate of patients with LM. It also decreases systemic side effects associated with chemotherapy. RIAC leads to higher remission rates because it directly targets the affected area. When RIAC is used alongside systemic chemotherapy, tumor resistance is decreased, increasing the rates of remission. This review aims to introduce the use of RIAC in patients with MBC. RIAC is a relatively new therapy in interventional oncology, and thus, limited research is currently available.

## Introduction and background

Breast cancer (BC) is a common malignancy affecting millions of women. Metastatic breast cancer (MBC) remains incurable despite new therapies that focus on molecular targeting [1-3]. Diagnosing BC before metastasis increases survival rates, but unfortunately, it is often diagnosed after metastasis [2]. It is known to have a poor prognosis because it is defined as a local disease that spreads to lymph nodes or organs quickly, impeding treatment approaches [2].

There are different subtypes of BC, which are grouped based on the immunohistochemical expression of the hormone receptors [4]. They each have different metastases, prognoses, and treatment approaches. The hormone receptors are estrogen receptor-positive (ER+), progesterone receptor-positive (PR+), and human epidermal growth factor receptor 2-positive (HER2+). When cancer is ER-negative (ER-), PR-negative (PR-), and HER2-negative (HER2-), it is called triple-negative breast cancer (TNBC) [3,5,6]. The higher the PR expression, the better the overall survival [2,4]. It is important to consider the relevance of HER2 in determining the treatment approach. HER2+ diagnoses are correlated with detrimental clinical outcomes because HER2 increases proto-oncogenic signaling pathways, leading to uncontrolled cancer cell growth [2,4]. It is present in 10%-15% of BCs. It requires specific drugs, such as trastuzumab, trastuzumab combined with emtansine, and pertuzumab, and tyrosine kinase inhibitors, such as lapatinib and neratinib, in addition to chemotherapy and surgical resection [4]. TNBC makes up 20% of all BCs and is typically diagnosed after it is in its advanced stage, so targeted chemotherapy may increase survival.

Currently, the median survival of patients with MBC is two years [1]. The five-year survival rate for MBC is 26% [3]. There are many therapeutic interventions to improve the quality of life and prolong life in patients experiencing MBC; however, it is difficult to target the affected area because each individual presents with different clinical scenarios. A patient can present with solitary metastatic lesions or multiple organ involvement [1]. When patients have a few metastatic lesions to a single organ, known as oligometastases, a multidisciplinary approach along with chemotherapy can improve survival [1]. Chemotherapy is the first-line therapy for MBC, especially in ER+ disease, with further use of hormonal agents and receptor-negative rapidly progressing disease [7]. The goals of chemotherapy are to prolong survival, prevent symptoms arising from the presence of the tumor, and improve quality of life; however, it does not cure the patient [8]. Approved drugs for primary BC include paclitaxel, belinostat, tocilizumab, 5-azacitidine, doxorubicin, cyclophosphamide, vorinostat, tazemetostat, atezolizumab, and methotrexate. Approved pharmaceutical therapies for MBC include gemcitabine, capecitabine, tivozanib, trastuzumab, lapatinib, pertuzumab, epirubicin, and palbociclib [3,5-7,9]. While chemotherapy is used to alleviate symptoms, it carries the risk of many toxicities such as significant fatigue, nausea, vomiting, diarrhea, hair loss, neutropenia, and neuropathy [8]. Further, patients with liver metastases (LM) tend to have a poor response [6]. LM develop in over half the women with MBC, are associated with poor prognosis, and indicate metastases to other sites [6].

Currently, the recommended treatment approach for MBC patients involves a multidisciplinary assessment of pathology, biomarkers, and menopausal status. For hormone receptor-positive MBC, hormonal therapy alone is used. For life-threatening diseases, surgery is the definitive approach. For premenopausal patients, ovarian suppression, along with hormonal therapy, is recommended. When the disease is HER2+ or triple-negative, single-agent chemotherapy is not suggested, but a combination of multiple chemotherapeutic agents is recommended. For HER2+ MBC, trastuzumab, capecitabine, and lapatinib are the first-line agents. For germline BRCA1 and BRCA2 mutation-positive MBCs, hormonal therapy with chemotherapy is used [10].

Regional intra-arterial chemotherapy (RIAC) delivers a high concentration of anticancer drugs directly to the site of malignant tissue, decreasing systemic side effects as seen in systemic (intravenous [IV]) chemotherapy [4]. A study has been carried out to find whether regional hepatic arterial infusion (HAI), in conjunction with systemic chemotherapy, is a treatment option for colorectal cancer patients with LM, which shows potential in treating metastatic cancers and reducing systemic progression [1,4]. When used in combination with systemic chemotherapy, there have been clinical benefits that result in decreased mortality rates and reduced progression of the disease [1,4]. HAI alone has better outcomes in treating metastatic cancers than systemic chemotherapy. Currently, the use of HAI in treating BC with LM is being studied. This literature review aims to introduce the use of HAI chemotherapy in BC patients with LM.

## Review

Methods

Search Strategy and Selection Criteria

A literature search was performed on January 16, 2024, using the Embase, Medline ProQuest, and PubMed databases. The Preferred Reporting Items for Systematic Reviews and Meta-Analyses (PRISMA) statement by the Cochrane Collaboration, London, United Kingdom, was used in writing this systematic review. Only clinical trials, cohort studies, cross-sectional trials, and primary studies were included in the study conducted in the last 20 years (2004-2024). Review articles, articles that involved patients with resectable BC, non-English articles, and articles that did not utilize intra-arterial chemotherapy were excluded. These criteria were utilized to fulfill the objectives of this review.

Key Terms

The following key terms were used to search the articles: intra arterial chemotherapy, arterial chemotherapy, hepatic chemotherapy, breast cancer, metastatic breast cancer, and breast metastases. Databases were searched using the Boolean operators “AND” and “OR” as follows: ((“intra-arterial chemotherapy*” OR (“intraarterial chemotherapy*” OR “arterial chemotherapy*” OR “hepatic intra-arterial*” OR “hepatic chemotherapy*” OR “hepatic intraarterial*”)) AND (“breast cancer*” OR “metastatic breast cancer*” OR “metastatic*” OR “breast metastases*”)). The articles were subsequently screened again to select the ones that met the inclusion criteria mentioned earlier.

Evaluation Process

**Figure 1:**
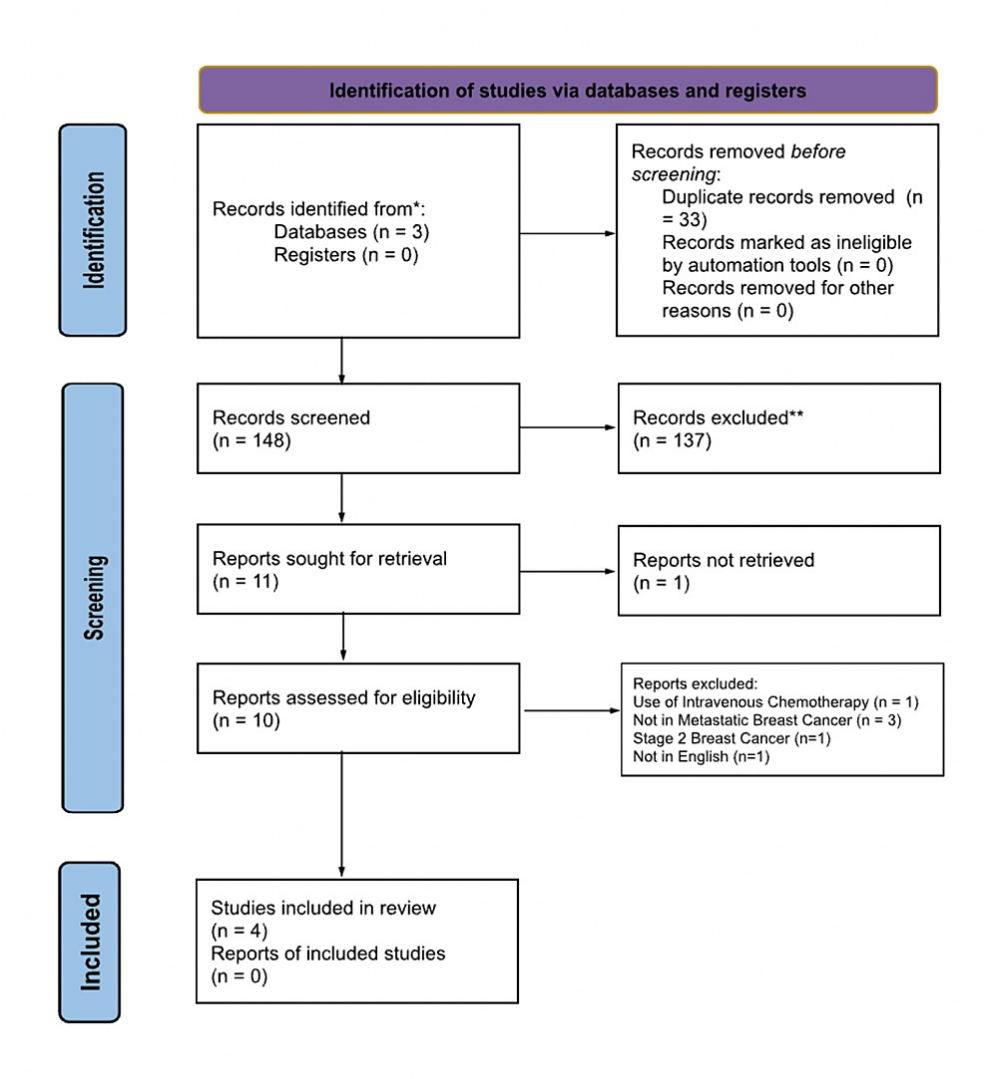
PRISMA diagram describing the process of article inclusion and exclusion. A total of 137 articles (after the removal of duplicates) were considered, among which four were found to be relevant to this study. PRISMA: Preferred Reporting Items for Systematic Reviews and Meta-Analyses *Usage of three databases: PubMed, Medline, and Embase **The number of records excluded by humans and not by automation tools

The process of how the articles were included was illustrated in the PRISMA diagram, as shown in Figure 1. Two of the authors (MB and HP) evaluated 148 unique articles after removing 33 duplicates. Based on inclusion and exclusion criteria, 137 articles were excluded, and 11 were sought for retrieval. Among them, 10 articles were obtained and evaluated to determine if they met the eligibility criteria. The authors (MB and HP) each independently reviewed the full-text publications. On further analysis, one article was excluded due to the use of IV chemotherapy, three were excluded due to only analyzing locally advanced BC, and one was excluded due to only analyzing stage 2 BC. Additionally, one article was excluded because it was in Japanese. Four studies were included after resolving disagreements through discussions with all authors. Additional reference lists were not utilized.

Data Collection

Each of the four articles was qualitatively evaluated. Once evaluated, a table containing the results was created with the following columns as shown in Table 1: reference, study type, sample size, aim, findings, limitations, and recommendations. The data were analyzed and the review was written based on the analysis.

**Table 1:** Articles included in the review HA: hepatic arterial; HAI: hepatic arterial infusion; BC: breast cancer; LM: liver metastases; CT: computed tomography; ER+: estrogen receptor-positive; PR-: progesterone receptor-negative; HER2-: human epidermal growth factor receptor 2-negative

Reference	Study type	Sample size	Aim	Findings	Limitations	Recommendations
Nielsen et al., 2012 [1]	Retrospective study	A total of 16 patients who received capecitabine daily along with oxaliplatin: seven patients received alternating intrahepatic and systemic oxaliplatin and nine patients had oxaliplatin HA, among whom five patients had only LM and 11 patients had LM and bone metastases	Evaluate the efficacy, activity, and toxicity through regional HAI of oxaliplatin with capecitabine in BC patients with LM	Moderate toxicity, progression-free survival of 7.9 months, and overall survival of 19.2 months	The number of patients and heterogeneous population; it cannot be excluded that patients might have received the same response with systemic chemotherapy	Increase the sample size and select diverse patient population
Ranieri et al., 2018 [11]	Case report	One patient	Evaluate effects of nab-paclitaxel systemic chemotherapy	HAI with 5-fluorouracil and nab-paclitaxel systemic chemotherapy, and LM completely disappeared on a CT scan	Only one patient	Evaluate the effects of nab-paclitaxel with HAI in a bigger sample size
He et al., 2011 [12]	Prospective study	A total of 47 triple-negative patients	Evaluate the efficacy of intra-arterial targeted chemotherapy in ER+, PR-, and HER2- BC	Intra-arterial chemotherapy is effective in treating triple-negative BC with the advantage of short-term course and good remission rates and prognosis	Only conducted in patients with triple-negative BC	Repeat in larger sample size and in patients with other types of BC
Camacho et al., 2007 [13]	Prospective study	A total of 10 patients with metastatic BC along with dominant LM. They received monthly 24-hour continuous hepatic infusions of paclitaxel through an intra-arterial catheter placed using a percutaneous transfemoral approach	Determine the safety and antitumor activity of hepatic intra-arterial paclitaxel therapy	A total of 56 courses of paclitaxel were delivered; the most common toxicities were leukopenia, fatigue, nausea, and vomiting; three patients had partial responses that lasted 6, 7, and 48 months; the other four patients had stable disease for five to nine months; one patient had liver resection after intra-arterial chemotherapy and remained disease-free for 48 months; no association between prior taxane exposure and efficacy of current regimen was established	Small sample size	Repeat in larger sample size and in patients with regional cancer

Results

Nielsen et al. conducted a retrospective study with 16 patients to evaluate the efficacy, activity, and toxicity of regional HAI in BC patients with LM. The main eligibility criteria were as follows: (1) radiological evidence of limited extrahepatic disease, (2) no bone lesions or regional lymph node progression within the past six months, (3) therapy with taxanes, (4) no prior therapy with capecitabine, and (5) at least one measurable lesion according to Response Evaluation Criteria in Solid Tumors guideline, version 1.1. Nine patients had a long-term percutaneous intra-arterial catheter implanted and connected to a subcutaneous infusion port. They received a dose of 85 mg/m^2^ of oxaliplatin administered over a period of 30 minutes biweekly. Seven patients received alternating intrahepatic oxaliplatin and IV oxaliplatin, each every fourth week. In the second group of patients, transarterial embolization was also administered for 12 cycles, not exceeding 1,000 mg/m^2^. Chemotherapy consisting of 1,300 mg/m^2^ of capecitabine daily was administered to both groups of patients. Patients with HER2+ tumors received additional treatment of 6 mg/m^2^ of trastuzumab triweekly. Antiemetics and analgesics were also administered at the discretion of the investigator. The median duration of therapy was 5.9 months (range: 2.7-12.1 months). Eight patients had a partial response, indicating an overall response rate of 50% (95% confidence interval, 95% CI: 25-75). One of these patients had an MRI scan showing a complete response and had no sign of progression 19 months after the termination of therapy. Eight patients had stable disease after six months (95% CI: 20-70). None of the patients showed a complete response immediately after HAI. No disease progression was observed in any patient after treatment was stopped. The median progression-free survival was 7.9 months (range: 3.2-36.0 months). The median overall survival was 19.2 months (range: 8.3-36.0 months). The most common toxicities noted were hand and foot syndrome in 37% of patients and sensory neuropathy in 50% of patients. Grade 3 neutropenia was observed in 13% of patients. No patients had thrombocytopenia. Grade 3 nausea and vomiting were seen in only one patient. Seven patients had grade 2 abdominal pain. Complications regarding catheter placement occurred in five patients: catheter-related thrombosis in one patient, infection in one patient, catheter displacement in one patient, and kinked catheter in two patients. No complications occurred in these patients once the catheter was replaced [1].

Ranieri et al. reported a case of a 55-year-old female who previously underwent a right radical mastectomy and lymph node dissection. Pathology showed stage 4, grade 3 infiltrating ductal carcinoma of the right breast with metastases. The patient received three lines of chemotherapy and one line of hormonal therapy. After one year, three LM were found. A fourth line of chemotherapy with capecitabine and vinorelbine was started, which was discontinued after one month due to toxicities. The decision was then made to try hepatic intra-arterial chemotherapy (HAI). HAI of 5-fluorouracil (1,200 mg/m^2^) dissolved in 100 mL of normal saline was performed for 48 hours in a continuous infusion every two weeks. Folinic acid (100 mg/m^2^ daily) was also added to the treatment regimen for two consecutive days every two weeks. Additionally, IV nab-paclitaxel 260 mg/m^2^ was administered triweekly. No serious toxicities were noted. The patient complained of grade 1 abdominal pain. After 16 cycles of HAI, all LM completely disappeared on computed tomography (CT), and CA-15.3 levels also decreased to the normal range. After multiple lines of treatment, a complete response was noted on the CT scan after the administration of HAI [11].

He et al. evaluated the efficacy of intra-arterial chemotherapy for the treatment of TNBC (ER-, PR-, and HER2-). Forty-seven patients with confirmed TNBC were enrolled in this study. Of these 47 patients, 19 had regional lymph node metastasis. All patients were treated with the cyclophosphamide, epirubicin, and fluorouracil (CEF) regimen (600 mg/m^2^ of cyclophosphamide, 90 mg/m^2^ of epirubicin, and 600 mg/m^2^ of 5-fluorouracil). Catheterization was performed; an infusion of 50% of the CEF regimen was given in the main feeding artery of cancer visualized under angiography, and 25% was administered into either the lateral thoracic or internal mammary artery followed by 25% administered between the distal end of the subclavian and vertebral arteries. The infusion lasted for three to five hours. Each cycle lasted for 21 days. The control group received an IV CEF regimen in a 21-day cycle. After completing one to two courses of chemotherapy, all patients underwent surgery. Six patients had a classical radical mastectomy, 10 had modified radical mastectomy, four had breast-conserving surgery, and four had palliative resection of BC. Within the control group, nine patients had a classical radical mastectomy, seven had a modified radical mastectomy, two had breast-conserving surgery, and five had palliative resection of BC. Additionally, 20 patients received radiotherapy [12].

In the patients who received targeted chemotherapy, changes were noticed as early as three days after administration. Short-term changes noted include decreased edema, improved superficial viscosity, decreased mass size, softened lesion, and reduced exudate of the ulcerated site. Nine patients achieved complete clinical remission, 13 patients had partial remission, one patient had stable disease, and four patients achieved complete pathological remission. The remission rate in this group was 91.67% (22 out of 24 patients). In the control group, three patients had complete clinical remission, 11 patients had partial remission, two patients had stable disease, and one patient had complete pathological remission. The remission rate in this group was 60.86% (14 out of 23 patients). In the 19 patients with axillary lymph node metastasis, no metastasis was found in the regional lymph nodes of seven patients after surgery. The efficacy of targeted chemotherapy was greater than standard IV chemotherapy (p = 0.018). Grade 0 toxicity was noted in 14 patients, grade 1 in seven patients, and grade 2 in three patients in the targeted chemotherapy group. Grade 0 toxicity was noted in 12 patients, grade 1 in seven patients, and grade 2 in four patients in the control group. No significant difference in drug toxicity was noted between the two groups (p > 0.05). Grade 0-1 toxicity resolved spontaneously, and grade 2 resolved one week after treatment ceased [4].

Camacho et al. conducted a prospective study to determine the safety and efficacy of HAI paclitaxel therapy in BC patients with predominant LM. Ten patients received monthly, 24-hour, continuous hepatic infusions of 200 mg/m^2^ of paclitaxel through an intra-arterial catheter, placed using the percutaneous transfemoral approach. This was repeated every four weeks until tumor progression, consent withdrawal, or until severe toxicities were noted. Patients had received a median of four prior systemic treatments with cytotoxic agents or taxanes. Eight patients previously had received taxanes as adjunctive therapy. The other two patients had not received taxane therapy. Leukopenia was the most observed side effect in eight patients, and nausea and vomiting were reported in eight patients. Two patients experienced rapid disease progression after one treatment course. One patient developed tumor progression after the third infusion and one after the fifth infusion. All four of these patients had marked progression in the liver (greater than 50%). The mean time of tumor progression from therapy initiation was 5.7 months. Three patients achieved a partial response. The mean duration of this response was 7.6 months. All patients eventually developed tumor progression. Due to the limited number of patients, no association between prior taxane exposure and benefit was noted [13]. The results of each study are shown in Table 1.

Discussion

RIAC administers higher concentrations directly to malignant tissue, reducing systemic side effects associated with IV chemotherapy. Although HAI therapy in Nielsen et al.’s study prevented disease progression in all BC patients with LM, there were no patients who had a complete response. However, disease progression did not occur, indicating that therapy with capecitabine and oxaliplatin may aid in prolonging the overall survival of patients. The toxicities present were hand and foot syndrome, sensory neuropathy, grade 3 neutropenia, abdominal pain, nausea, and vomiting [1]. However, the toxicities were not severe and were lower than those of patients who received systemic chemotherapy.

Ranieri et al. evaluated a case of a 55-year-old female with stage 4, grade 2 infiltrating ductal carcinoma of the right breast with metastasis who received three lines of chemotherapy and one line of hormonal therapy [11]. Even after treatment, the patient had two LM. After failing another chemotherapy regimen, 16 cycles of HAI with 1,200 mg/m^2^ of 5-fluorouracil in 100 mL of normal saline for 48 hours every two weeks were completed. A repeat CT scan showed no presence of LM, exhibiting the use of HAI chemotherapy in patients with LM. It was important to note that the patient did not experience any systemic effects of chemotherapy from HAI other than grade 1 abdominal pain [11]. The absence of negative symptoms and the successful resolution of LM with HAI chemotherapy indicated the positive use of HAI chemotherapy treatment.

In relation to TNBC, treatment with targeted chemotherapy resulted in symptom improvement as early as three days. Patients experienced decreased edema and mass size, reduced exudate at the ulcerated site, and a softened lesion [12]. The remission rate was 91.67% in patients who received targeted chemotherapy and 60.86% in the control group who were treated with the IV CEF regimen (600 mg/m^2^ of cyclophosphamide, 90 mg/m^2^ of epirubicin, and 600 mg/m^2^ of 5-fluorouracil) [12]. The significantly higher remission rate in targeted chemotherapy with HAI chemotherapy in comparison to systemic chemotherapy was an important finding to increase the survival rate in patients diagnosed with BC.

The safety and efficacy of HAI paclitaxel therapy were evaluated in the study by Camacho et al. Of the 10 patients evaluated, four patients had systemic taxane chemotherapy as an adjunctive therapy and another four patients had systemic taxane chemotherapy for metastases [13]. The three patients who had a partial response to HAI chemotherapy had systemic taxane chemotherapy prior to HAI chemotherapy. When HAI chemotherapy with either a high dose of paclitaxel or prolonged exposure with paclitaxel was present, alongside systemic chemotherapy, there was markedly decreased tumor resistance. The decreased presence of tumor resistance displayed the benefit of HAI chemotherapy over the IV route. Also, compared to systemic chemotherapy, HAI chemotherapy presented fewer systemic toxicities [13].

Patients treated with HAI chemotherapy, whether alone or in adjunct to systemic chemotherapy, had greater remission rates, lower systemic side effects, and improved overall survival. These clinically important findings displayed the use of HAI in BC patients with LM. HAI could be used as a targeted therapy to reduce systemic progression. As it is a recent advancement in interventional oncology, more research is needed to evaluate the significant outcomes of hepatic intra-arterial chemotherapy.

Limitations

It is crucial to consider this study's limitations in evaluating medical literature and analyzing results. It is possible that some primary studies may have been missed since only three databases were used. Additionally, non-English articles are excluded, which may have yielded other pertinent results. Another common limitation of these articles is the small sample size. Thus, it may be difficult to extrapolate this treatment regimen to a larger population.

Future Studies

Additional future studies should be conducted in larger sample sizes to better understand adverse effects and effective therapeutic doses and regimens. Studies should also be repeated in a more diverse population. Prospective studies may provide an added benefit in quantifying patient outcomes. Future studies focusing on comparing different chemotherapeutic regimens within the same patient population may also be useful.

## Conclusions

BC is a common malignancy in women. There are many subtypes of BC, which are grouped based on hormone receptors ER+, PR+, and HER2+. TNBC is diagnosed when all three hormone receptors are negative. MBC is incurable even with the development of new therapies that target the hormone receptors. Currently, systemic chemotherapy is the first-line treatment in BC with metastases. However, systemic chemotherapy has a poor response when LM are present, which are found in over half the women with MBC. RIAC delivers higher concentrations of chemotherapy directly to the malignant cells and decreases systemic adverse effects that are present in IV chemotherapy. This review aims to introduce the use of HAI chemotherapy for the treatment of BC metastases. This relatively new treatment approach has to be further developed to evaluate its impact on metastatic cancers.
